# Editorial: The impact of genetics on CRC therapy: from adaptive mutability to drug resistance

**DOI:** 10.3389/fonc.2023.1260158

**Published:** 2023-08-08

**Authors:** Giovanni Crisafulli, Giulia Siravegna

**Affiliations:** ^1^ IFOM ETS - The AIRC Institute of Molecular Oncology, Milan, Italy; ^2^ Vidium Animal Health, Scottsdale, AZ, United States

**Keywords:** cancer evolution, genetics, genomics, drug resistance, target therapies, adaptive mutability, CRC (colorectal cancer)

Colorectal cancer (CRC) stands as one of the most common and lethal malignancies, contributing to approximately 9% of cancer-related deaths and 10% of all new cancer diagnoses worldwide every year (1).

The genetic landscape of CRC is characterized by a remarkable level of heterogeneity, which has been exploited to stratify patients into distinct tumour classes (2–4). In the era of precision oncology, the accurate stratification of patients based on their tumor’s genetic features is crucial to guide effective therapeutic interventions (5). Even when targeted therapies show to be effective in achieving an initial, partial response to the treatment causing tumour shrinkage, this response is often transitory (6–10). Indeed, after some time, resistance to targeted therapies occurs in virtually all cases (11–17).

Tumors may harbor subpopulations of drug-resistant cells, which can survive under the selective pressure of the drugs regimen. The existence of pre-existing resistant clones poses a significant hurdle in achieving durable responses, underscoring the urgent need for the discovery of novel strategies focused on promptly identifying and overcoming resistance right from the initiation of treatment (11–17). In addition to the presence of pre-existing resistant clones, CRC tumour can also generate *ex-novo* resistance clones through mechanisms of adaptive mutability (18–20) (**Figure 1**).

**Figure 1:**
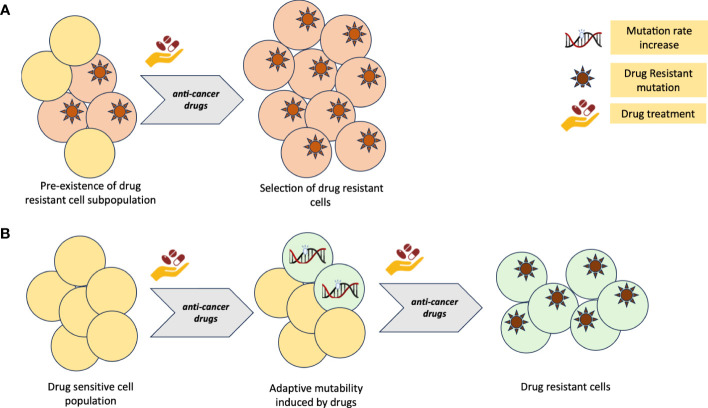
**(A)** Primary resistance to cancer therapy model due to pre-existing, drug insensitive tumour clones. **(B)** Adaptive mutability model of acquired resistance to cancer therapy. Figure created with BioRender.com

It has been shown (21) that, under therapeutic pressure, tumour cells exhibit increased mutational rates, thus facilitating the emergence of novel genetic alterations responsible for drug resistance. This adaptive response allows the tumour to evade the effect of the targeted therapy and continue proliferating, ultimately leading to treatment failure (18, 19, 21). Identifying the underlying mechanisms driving this adaptive mutability process is crucial to develop effective strategies able to counteract the emergence and selection of resistant clones.

In this special Research Topic, entitled “*The impact of genetics on CRC therapy: from adaptive mutability to drug resistance*” the authors have focused on the role that the CRC genetics plays on the therapy efficacy. Four research studies (Shailes et al.; Qin et al.; Luo et al.; Cai et al.), included in this special Research Topic, have shed light on different aspects of colorectal cancer’s pathogenesis and provided insights into potential therapeutic strategies, prognostic markers, and molecular mechanisms involved in the disease progression and treatment response of CRC.

Shailes et al. focused on identifying therapeutic strategies for the treatment of CRC carrying *APC* mutations, which account for nearly 80% of all CRC cases and are also responsible for the inherited form of CRC (26, 27). They employed an *APC* mutant CRC cell line and CRISPR-cas9 gene editing approach to perform an FDA-approved drug screen targeting over 1000 compounds. As a result, they identified a statin, the HMG-CoA reductase inhibitor, as a potential therapeutic agent in APC mutant CRC. Statins have been demonstrated to induce a significant loss in cell viability specifically in *APC* mutant cell lines and patient-derived xenograft models. Importantly, Shailes et al. highlighted the synthetic lethal relationship between *APC* mutations and the treatment with statin, which could be further explored to treat other *APC* mutated cancer types, such as thyroid, brain cancer and pancreatic cancers (22). Identifying robust biomarkers and developing novel technologies for early detection of resistance-associated genetic alterations may enable prompt modification of therapeutic regimens, ensuring better patient outcomes.

Within this context, the objective of the study of Qin et al. was to investigate the association between genetic variations in genes involved in the immune regulation signaling and the clinical outcomes in metastatic colorectal cancer (mCRC) patients treated with bevacizumab-based chemotherapy, a well-established first-line treatment in this setting. The authors genotyped specific single-nucleotide polymorphisms (SNPs) by using DNA from blood samples of 141 mCRC patients and identified the “AA” genotype of rs2297136 in the *CD274* gene to be correlated with better progression-free survival (PFS) and increased overall survival (OS), as opposed to patients harboring the “AG” or “GG” genotypes. These findings suggested that rs2297136 could serve as an important predictor for mCRC patients’ prognosis in the context of bevacizumab-based chemotherapy.

Another example of the importance of genetics for patients’ stratification was reported by Luo et al. This study aimed at developing a prognostic model based on cuproptosis-related genes in colon adenocarcinoma (COAD). Cuproptosis is a specific type of cell death induced by an overload of copper ions in the cell (28). Through extensive data analysis of The Cancer Genome Atlas (TCGA) (2) and the Gene Expression Omnibus (GEO) databases, the authors generated a risk model comprising of five cuproptosis-related genes (CKDN2A, SDHB, CCS, ULK1, and CMC1), after screening for 30 differentially expressed genes from patients with COAD. Based on this model, in both cohorts, the low-risk group exhibited longer overall survival. The study also showed how changes in immune cells within the tumour microenvironment as a potential mechanism underlying the prognostic implications of the risk model.

In the last study published in the Research Topic, Cai et al. reported an integrative analysis investigating the role of pyroptosis-related long non-coding RNAs (PRLs) in CRC. Pyroptosis represents a form of cell death that is triggered by proinflammatory signals and associated with inflammation (29). Using transcriptomic data from TCGA (2) and external datasets, they constructed a risk model comprising eight PRLs (Z99289.2, FENDRR, CCDC144NL-ASL, TEX41, MNX1-AS1, NKILA, LINC02798, and LINC02381*).* These PRLs were stratified into two molecular subtypes involved in immune modulators, immune infiltration of tumor immune microenvironment, and inflammatory pathways, and three independent methods were applied to predict PRL-related sensitivity to chemotherapeutic drugs. This, together with the ability of predicting treatment response and prognosis, are major contributors to a better understanding of CRC overall pathogenesis and the development of effective immunotherapy regimens.

In summary, it is now well acknowledged how the understanding of the genetic mechanisms underlying drug resistance is fundamental to improve precision oncology strategies in CRC patients. Among other cancer types, CRC presents one of the most complex genetic landscapes, characterized by highly heterogeneous tumour subpopulations, making it even more challenging to find the optimal targeted therapy and extend overall survival. Advancing our understanding of the mechanisms driving the emergence and selection of resistance clones through specific mechanisms of cell death [pyroptosis or cuproptosis which are apparently different from apoptosis, necroptosis, and ferroptosis (30)] is crucial to develop effective precision oncology strategies, as addressed by this special Research Topic. Further investigations and validations are warranted to translate these findings into clinical practice, with the ultimate goal of improving patient’s outcome.

In this special Research Topic, the authors considered the impact of genetics and transcriptomics in CRC. Further studies should focus on the integration of genomic profiling with other omics, immune infiltration of tumor microenvironment and the dynamic monitoring of tumour evolution during novel combination therapies, all of which hold promises in fighting the emergence of resistance and maximizing treatment efficacy. Furthermore, the study of adaptive mutagenesis in the context of colorectal cancer therapy is still in its early stages. However, the potential to develop novel drugs that could target the modulation of mutational rates is promising. By impeding the cancer cells’ adaptive capability, we can potentially prevent the emergence of resistant clones and pave the way for more effective and durable treatment strategies. Continued research and exploration of this field are vital for advancing our understanding and transforming the landscape of colorectal cancer therapy.

## Author contributions

GC: Conceptualization, Resources, Supervision, Writing – original draft, Writing – review & editing. GS: Resources, Supervision, Writing – original draft, Writing – review & editing, Conceptualization.
